# The Association between Vitamin D and Anti-Müllerian Hormone: A Systematic Review and Meta-Analysis

**DOI:** 10.3390/nu12061567

**Published:** 2020-05-28

**Authors:** Irene Moridi, Alice Chen, Oded Tal, Reshef Tal

**Affiliations:** 1 Division of Reproductive Endocrinology & Infertility, Department of Obstetrics, Gynecology and Reproductive Sciences, Yale University School of Medicine, New Haven, CT 06510, USA; ir_moridi@yahoo.com (I.M.); Alice.y.chen@yale.edu (A.C.); 2 Department of Obstetrics & Gynecology, Icahn School of Medicine at Mount Sinai, Queens Hospital Center, New York, NY 10029, USA; 3 School of Business and Hospitality, Conestoga College, Kitchener, ON N2G 4M4, Canada; otal@conestogac.on.ca

**Keywords:** vitamin D, anti-Müllerian hormone (AMH), ovarian reserve, fertility, systematic review

## Abstract

Accumulating evidence from animal and human studies indicates a role for vitamin D in female reproductive physiology, and numerous clinical studies have suggested its potential benefit for various aspects of human reproduction. Anti-Müllerian hormone (AMH) is an ovarian biomarker that plays an important role in folliculogenesis. It is the most sensitive ovarian reserve marker and is widely used clinically in reproductive medicine. While initial studies have suggested that vitamin D may be associated with ovarian reserve markers, including AMH, evidence has been conflicting. Currently, there is considerable debate in the field whether vitamin D has the capacity to influence ovarian reserve, as indicated by the AMH level. The current systematic review aims to evaluate and summarize the available evidence regarding the relationship between vitamin D and AMH. In total, 18 observational studies and 6 interventional studies were included in this systematic review. Cross-sectional studies have reported largely discrepant findings regarding an association between serum vitamin D and AMH levels, which are likely due to the heterogeneity in study populations, as well as the apparently complex relationship that may exist between vitamin D and AMH. However, meta-analysis of interventional studies performed herein that examined the effects of vitamin D supplementation on serum AMH levels indicates a cause-effect relationship between vitamin D and AMH, the direction of which appears to depend on a woman’s ovulatory status. Serum AMH was significantly decreased following vitamin D supplementation in polycystic ovarian syndrome (PCOS) women (standardized mean difference (SMD) −0.53, 95% CI −0.91 to −0.15, *p* < 0.007), while it was significantly increased following vitamin D supplementation in ovulatory women without PCOS (SMD 0.49, 95% CI 0.17 to 0.80, *p* = 0.003). In conclusion, the results of this systematic review demonstrate that the relationship between vitamin D and AMH is a complex one, and large, randomized trials of vitamin D supplementation focusing on different vitamin D status ranges are necessary to gain more insight into the nature of this relationship and the potential benefit of vitamin D to female reproduction in general.

## 1. Introduction

Vitamin D is a steroid hormone, mainly produced by the skin upon exposure to sunlight, with less than 20% supplied by dietary sources [1,2]. Hydroxyvitamin D (25OH-D) is produced by the conversion of vitamin D by hepatic 25-hydroxylase, and the active form 1,25-dihydroxyvitamin D3 is made by the action of 1a-hydroxylase, primarily in the kidney. Local synthesis of the active form of vitamin D occurs by 1a-hydroxylase also in various other tissues such as ovaries, breasts, prostate, brain, and colon. The main function of vitamin D is the metabolic regulation of calcium and phosphate homeostasis, thus controlling the quality of bone mineralization, which is essential for the prevention of rickets in children and osteomalacia or osteoporosis in adults [3]. The vitamin D receptor (VDR) is a member of the steroid/thyroid nuclear hormone receptor superfamily [1,4]. VDR is found not only in classical tissues known to regulate calcium metabolism such as the intestines, skeleton, and parathyroid glands but, also, in reproductive organs such as the ovaries, uterus, placenta, testes, hypothalamus, and pituitary [5,6,7,8]. Animal studies showed that VDR is expressed in cycling mice in reproductive tissues, including endometrium, ovaries, and fallopian tubes, and it is expressed in the placenta and decidua in pregnant mice [9,10], suggesting an important role for vitamin D in reproductive physiology. Recently, other roles such as antiproliferation, anti-inflammation, prodifferentiation, proapoptosis, and immunosuppression have been attributed to vitamin D [1,5] and have led to consideration of vitamin D as a potential treatment for diseases such as diabetes, psoriasis, cancer, and immune disorders, among others [11,12,13].

Accumulating evidence from both animal and human studies suggests a role for vitamin D in female reproductive physiology [14] although little is known about the underlying mechanism. Dietary vitamin D deficiency results in a decrease of overall fertility in rats by 75%, associated with a 30% reduction in litter size and impaired neonatal growth [15]. VDR knockout mice showed impaired bone formation and growth retardation, uterine hypoplasia, and impaired folliculogenesis [16,17]. These animals exhibited hypergonadotropic hypogonadism accompanied by decreased aromatase activity and CYP19 gene expression, pointing to the potential role for vitamin D in estrogen biosynthesis and aromatase gene expression [16,17]. In mice harboring a knockout in 25-hydroxyvitamin D 1a-hydroxylase, similar defects, including uterine hypoplasia, impaired follicular development, and defective corpus luteum formation, are observed. Importantly, treatment with high calcium and/or phosphorus diet restores the fertility of these animals, suggesting that infertility is secondary to hypocalcemia and/or hypophosphatemia caused by vitamin D deficiency in these models [18,19,20]. In human studies, the in vitro treatment of ovarian cells with vitamin D increased production of the sex-steroids progesterone, estrogen, and estrone [21,22]. Moreover, 1,25-dihydroxyvitamin D3 stimulated estrogen and progesterone production in the human placenta [23]. In addition, a mounting body of evidence suggests that vitamin D deficiency is associated with various manifestations of polycystic ovarian syndrome (PCOS), including anovulation, hyperandrogenism, and insulin resistance [24]. Importantly, vitamin D deficiency is more common in PCOS women [25,26], and vitamin D supplementation has been shown to improve menstrual cyclicity, hyperandrogenism, and various metabolic aspects of this syndrome [27,28,29], indicating a direct beneficial effect of vitamin D on female fertility.

One of most important biomarkers produced by the granulosa cells, which plays an important role in folliculogenesis, is the anti-Müllerian hormone (AMH). AMH is a member of the transforming growth factor-beta superfamily, also known as the Müllerian-inhibiting substance (MIS) [30]. It is involved in the regression of the Müllerian ducts in mammalian and avian male embryos [31,32,33]. In the human female, its production begins only after the 36th week of gestation, and it is secreted exclusively by granulosa cells of ovarian follicles independent of gonadotropins [34]. Its secretion begins when follicles are recruited from the primordial pool to become primary follicles, peaks at the preantral/small antral stage, and diminishes when they reach the final size and differentiation state available for selection by the pituitary follicle-stimulating hormone (FSH) [30]. AMH regulates the development of early preantral and small antral follicles in mice, whereas its effects on follicular recruitment in primates and humans are less clear-cut. It acts as an inhibitor of FSH sensitivity of growing antral follicles, an effect that is not species-specific [35]. While some studies noted significant fluctuations of AMH within one menstrual cycle [36,37], AMH is considered to be relatively stable throughout the menstrual cycle [34]. The relatively small fluctuations of serum AMH during the menstrual cycle, a strong correlation with the primordial oocyte pool size, as well as the follicular response to ovarian stimulation, makes it superior to other ovarian reserve markers (such as day 3 FSH and day 3 inhibin), as well as clinically useful and convenient for patients [34]. There are pathological situations, however, when AMH levels do not correlate with the ovarian reserve. For instance, in PCOS and hypothalamic amenorrhea, AMH levels may be abnormally high and abnormally low, respectively, which reflect the specific stage of arrest in follicular development rather than the size of the primordial follicular pool.

At present, there is considerable debate in the field regarding whether vitamin D has the capacity to influence ovarian folliculogenesis, as indicated by the AMH level, as well as what direction that influence may take. This question is of particular importance given the increasing attention that vitamin D supplementation has received lately from the reproductive medicine community based on accumulating evidence that vitamin D may be beneficial for fertility and pregnancy. To date, there have been numerous observational studies and a few interventional studies aimed at evaluating the relationship between serum vitamin D and AMH levels, yielding largely conflicting results. The purpose of the present systematic review is to evaluate and summarize the current literature regarding the relationship between vitamin D and AMH as an ovarian reserve marker.

## 2. Materials and Methods

### 2.1. Search Strategy, Study Selection, and Data Extraction

A systematic literature review was conducted in PubMed, Embase, Web of Science, Cochrane Library, and ClinicalTrials for relevant publications in English through February 2020 to identify prospective and retrospective clinical studies assessing the relationship between vitamin D and AMH. The study was conducted according to PRISMA guidelines for the reporting of systematic reviews and meta-analyses. It was registered in the International Platform of Registered Systematic Review and Meta-analysis Protocols (INPLASY) with registration number INPLASY202040204. The searches included combinations of the following MESH and non-MESH terms: “vitamin D”, “25 hydroxyvitamin D”, “AMH”, “anti-Müllerian hormone”, “MIS”, “Müllerian inhibiting substance”, and “ovarian reserve”. Bibliographies were cross-referenced to identify additional studies. The searches were conducted independently by I.M. and A.C. Disagreements were resolved by discussion and consensus among I.M., A.C. and R.T. If a study fulfilled the eligibility criteria, it was included in the systematic review. For the vitamin D interventional studies, if information was available regarding pretreatment and posttreatment AMH and vitamin D levels, the study was selected for final inclusion in the meta-analysis. Data were extracted from the articles text and tables and organized into tables in a systematic manner. The following information was extracted: the last name of the study’s first author, country, study population and sample size, inclusion and exclusion criteria, intervention (if applicable), serum AMH levels, serum vitamin D levels, the relationship between AMH and vitamin D, and covariates adjusted.

### 2.2. Eligibility Criteria

Studies were included in this systematic review if they met the following criteria: (1) the study population included reproductive-age women, (2) serum AMH and vitamin D were measured in all study participants concomitantly in at least one time point, (3) the association between serum vitamin D and AMH levels was described and quantitative information was provided, and (4) any study design except case reports. Thus, studies referring to follicular fluid AMH or vitamin D were excluded. In addition, studies referring to males or prepubertal or menopausal women were excluded.

### 2.3. Study Quality Assessment

Each interventional cohort study selected for final inclusion in the meta-analysis was scored by the researchers (A.C. and R.T.) using the Newcastle-Ottawa scale (NOS) regarding the following study quality characteristics: (1) representativeness of exposed cohort, (2) selection of nonexposed cohort, (3) exposure assessment, (4) outcome of interest not present at the start of the study, (5) comparability of cohorts, (6) outcome assessment, (7) adequacy of length of time before follow-up, and (8) adequacy of follow-up of cohorts.

### 2.4. Data Analysis

Continuous measures meta-analysis was conducted using MedCalc version 19.2.0 (MedCalc Software) using the standardized mean difference (SMD) to compare the various studies. Heterogeneity among studies was quantified by Cochran’s Q test and I-squared measure; *p* < 0.10 and I^2^ > 50% indicates statistical heterogeneity. The effect of vitamin D supplementation on vitamin D levels across all 6 cohorts was calculated using the random effects model. The effect of vitamin D supplementation on AMH levels across all 6 cohorts was calculated using the random effects model. Since the effect on the AMH level in 3 studies related to PCOS patients was the opposite of the effect in 3 studies related to non-PCOS patients, two additional meta-analyses were conducted on each of these subgroups. In both cases, the effect of vitamin D supplementation was calculated using the fixed effects model. This is based on the assumption that the studies share a common true effect: increasing AMH levels in non-PCOS patients and decreasing AMH levels in PCOS patients.

## 3. Results

### 3.1. Search Results

**Figure 1:**
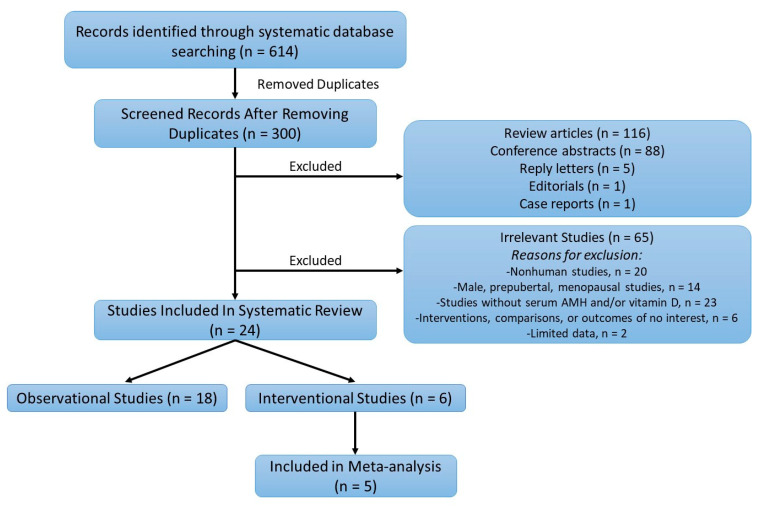
Flowchart of the systematic literature search strategy and results. AMH: anti-Müllerian hormone.

The systematic literature search retrieved 614 articles. Figure 1 summarizes the number of publications that were identified during the initial search and the number of publications that were finally selected for inclusion after appropriate exclusion. After removing 300 duplicates, and 211 studies that were not original cohort articles, 65 articles were excluded by screening the title and abstract, and then, the remaining 24 articles were inspected carefully for eligibility. In total, 18 observational studies and 6 interventional studies were selected for the systematic review. Among the six interventional studies, five included the relevant information of pre- and posttreatment AMH and vitamin D values and were selected for data extraction and meta-analysis. The quality assessment of the studies selected for the meta-analysis is shown in Supplementary Table S1.

### 3.2. Evidence for a Relationship between Vitamin D and AMH at the Molecular and Cellular Level

The effect of vitamin D on AMH gene expression was first reported by Krishnan et al. [38], who showed an upregulation of AMH mRNA with calcitriol treatment in vitro in a human prostate cancer cell line. The same group later identified a functional vitamin D-response element (VDRE) in the human AMH promoter region, providing a direct molecular link for vitamin D effects on AMH gene expression [39]. However, an in vitro study in a hen granulosa cell culture by Wojtusik et al. [40] found the opposite effect, that 1,25-dihydroxyvitamin D3 decreased the expression of AMH mRNA levels. This was associated with increased FSH receptor expression and cell proliferation, suggesting a positive role for vitamin D in follicular development and selection through increasing the FSH receptor and decreasing the inhibitory action of AMH. In a primate study, three-dimensional cultures were established from secondary preantral follicles isolated from rhesus macaque ovaries. Although progesterone and estrogen production by antral follicles was not altered by vitamin D3, AMH concentrations were 36% higher in the vitamin D group relative to controls, and vitamin D3 treatment increased preantral follicle survival and improved antral follicle diameters, consistent with a direct positive effect upon ovarian folliculogenesis [41]. In another study by Merhi et al. on women undergoing ovarian stimulation for in vitro fertilization (IVF), there was a two-fold increase in AMH receptor II (AMHR-II) expression in granulosa cells of women with insufficient/deficient follicular fluid 25OH-D (<30 ng/mL) compared with those with normal follicular fluid 25OH-D levels (>30 ng/mL) [21]. In that study, the treatment of human luteinized cumulus granulosa cells with vitamin D3 resulted in a significant decrease in AMHR-II and suppression of the AMH-induced SMAD 1/5/8 phosphorylation [21]. These findings suggest that vitamin D can counteract the inhibitory effect of AMH on granulosa cell differentiation and follicular growth by inhibiting AMHR-II expression and downstream signaling. However, the terminally differentiated (luteinized) granulosa cells obtained from mature follicles after controlled ovarian hyperstimulation in that study may be markedly different from granulosa cells of developing follicles. While the results of the above studies are somewhat conflicting in terms of the effects of vitamin D on the direction of AMH gene expression, it is clear that vitamin D can influence AMH gene expression and downstream signaling, an effect likely mediated directly via the VDRE on the AMH gene promoter.

### 3.3. Systematice Review

#### 3.3.1. Observational Studies

The data from 18 cross-sectional studies is summarized in Table 1. In 2012, Merhi et al. [42] examined the relationship between serum vitamin D and serum AMH levels in US women enrolled in the Women’s Interagency HIV Study (WIHS). This cross-sectional study included 388 HIV-positive and risk-matched HIV-negative women with normal menstrual cycles. It was demonstrated in a multivariate linear regression analysis that serum 25OH-D was positively correlated with serum AMH levels in late-reproductive-age women (>40 years old; regression slope +0.011; *p* = 0.028) after adjusting for various confounders. Interestingly, a weak negative correlation between serum vitamin D and AMH was noted in young individuals (<35 years of age; *r*^2^ = −0.21, *p* = 0.019, regression slope; *r*^2^ = −0.0086, SE = 0.004, *p* = 0.054), which was rendered insignificant after adjustment for the same covariates. Other research groups have found no correlation between vitamin D and AMH serum levels. In 2014, Chang et al. [43] conducted a cross-sectional study of 73 healthy Korean women. After adjusting for age and body mass index (BMI), they found a positive relationship between 25OH-D and free testosterone, total testosterone, and the free androgen index. Vitamin D did not correlate with AMH (*r* = 0.001, *p* = 0.99), FSH, the antral follicle count (AFC), and the ovarian volume.

In a larger study from Southern Australia, Pearce et al. [49] examined the relationship between serum vitamin D and AMH levels across the four seasons in women presenting to a private fertility clinic. Major strengths of this study are its large sample size, as well as separately analyzing ovulatory women (*n* = 282) and PCOS women (*n* = 58). As expected, vitamin D levels exhibited variations across the four seasons. However, AMH levels showed no correlation with vitamin D levels over the year (*r*^2^ = 0.04, *p* = 0.4), even after adjustment for known confounders such as age and BMI (*r*^2^ = 0.04, *p* = 0.5). When the cohort was divided into PCOS and ovulatory groups, still no significant relationship was observed (*r*^2^ = −0.014, *p* = 0.96 PCOS; *r*^2^ = 0.094, *p* = 0.11 ovulatory). Moreover, serum vitamin D was not related to AFC in the overall cohort (*r*^2^ = 0.03, *p* = 0.85) or in those individuals with or without PCOS, even after adjustment for covariates.

There have been other studies that specifically evaluated the relationship between vitamin D and AMH in PCOS women. A study on PCOS women by Bakeer et al. [51], which included 53 infertile PCOS women and 17 healthy ovulatory women, similarly showed no significant correlation between 25OH-D and AMH in either the PCOS or control group (r = −0.303). In contrast, in a large study from Hong Kong that included 451 PCOS women and 244 ovulatory women, Wong et al. [50] found that serum 25OH-D levels in PCOS women significantly correlated positively with AMH, the AMH/AFC ratio, and other metabolic parameters. They found that the 25OH-D level was an independent predictor of the serum AMH level after controlling for age, BMI, and the free androgen index in women with PCOS but not in ovulatory women [50]. More recently, a study by Szafarowska et al. that examined genetic polymorphisms in the vitamin D receptor (VDR), AMH, and AMHR-II genes in PCOS women also reported an association between vitamin D and AMH levels [52]. The study included seventy-five patients with PCOS and 23 control ovulatory women and found no correlation between serum AMH and 25OH-D levels. Interestingly, the study found an association between VDR polymorphisms and AMH levels in PCOS women, while there was no correlation between AMH and AMHR-II gene polymorphisms and AMH levels [52]. Arslan et al. [53] similarly found no correlation between serum AMH and 25OH-D levels in PCOS patients (r = 0.027, *p* = 0.836) and control individuals (r = −0.112, *p* = 0.307). This study included 146 Turkish infertile women that were divided into normal ovarian reserve patterns (*n* = 86) and PCOS ovarian reserve patterns (*n* = 60). Within these two groups, women were further divided into severe (<10 ng/mL, Group A) and mild (10–20 ng/mL, Group B) vitamin D deficiency. There were no significant differences in vitamin D levels between the normal and PCOS groups.

Other studies focusing on healthy noninfertile reproductive-age women also did not find a clear association between vitamin D and serum AMH levels. Fabris et al. [44] analyzed a retrospective cohort of 851 healthy young oocyte donors with regular menstrual cycles from Spain and failed to show a correlation between serum-bioavailable vitamin D and either serum AMH levels (r = 0.059) or AFC (r = 0.081). In a US study by Jukic et al. [46] that examined an older cohort of reproductive-age women who were trying to conceive, 25OH-D was not found to be correlated with AMH, FSH, or inhibin-B (*r* < 0.03 for all). Multivariable results with continuous hormonal outcomes were also null. However, for dichotomous outcomes, there was a tendency for insufficient 25OH-D (<30 ng/mL) to be associated with low AMH (<0.7 ng/mL) (odds ratio (95% CI): 1.8 (0.9–4)). An earlier study by the same group of 527 premenopausal women also suggested an association between the vitamin D level and FSH [60]. They showed an inverse association between 25OH-D and urinary FSH. The median 25OH-D level was 12 ng/mL, with approximately 75% of participants below the recommended level of 20 ng/mL. For an increase of 10 ng/mL in 25OH-D, urinary FSH decreased by 14% (95% confidence interval: −23%–−5%), *p* = 0.003 [60]. A study of Korean women of similar reproductive ages by Kim et al., however, did not find a correlation between serum vitamin D and AMH levels [45]. The investigators evaluated the relationship between AMH and 25OH-D and the metabolic syndrome (MetS) risk in 291 healthy, late-reproductive-age (35–49 years) women with regular menstrual cycles. They conclude the serum AMH level decreased with age, but there is no correlation between AMH and vitamin D or MetS risk components in late-age reproductive women who had regular menstrual cycles [45]. A large US study that analyzed data from 656 late-reproductive-age female nurses who participated in the Nurses’ Health Study II (NHS2) prospective study was conducted by Purdue-Smithe et al. [47], also showing that serum AMH did not vary with total 25(OH)D levels across four vitamin D quartiles. It included women who experienced menopause between the time of blood collection and age 45 (cases, *n* = 328) and women who experienced menopause after age 48 (controls, *n* = 328). Interestingly, AMH geometric mean concentrations did vary with vitamin D-binding protein (VDBP) concentrations (*p* = 0.04). Strengths of this study included the use of the ultra-sensitive Pico AMH assay, as well as its large sample size, which allowed cases (women who experienced menopause before age 45) to be matched 1:1 with controls (women who experienced menopause after age 48) according to age and other factors. In a study that included women diagnosed with premature ovarian insufficiency (POI) and control noninfertile women, Xu et al. [48] found no difference in 25(OH)D levels between women with POI (*n* = 33) and control women (*n* = 72) (*p* = 0.523). In addition, while there was a positive relationship between 25(OH)D levels and log-transformed AMH, this correlation was not statistically significant (r = 0.175, *p* = 0.075), even when adjusted for age, BMI, education, and household income (r = 0.153, *p* = 0.120).

Several cross-sectional studies were conducted on populations of women with infertility, yielding largely negative results for an association between serum vitamin D and AMH concentrations. A study from Belgium by Drakopoulos et al. [55] examined the association between serum vitamin D and ovarian reserve markers in 283 infertile women, the majority of which were Caucasian, undergoing their first fertility treatment. This study did not find any association between vitamin D and AMH or AFC; vitamin D-deficient women (<2 0 ng/mL) had similar serum AMH (3.9 ± 3.8 vs. 4.3 ± 4.8 ng/mL, *p*= 0.5) and AFC (13.9 ± 1 3.3 vs. 12.7 ± 1 1.4, *p* = 0.7) compared to women who were not deficient (≥2 0 ng/mL), respectively [55]. Another cross-sectional study by Shapiro et al. was performed on 457 infertile US women with a high prevalence of diminished ovarian reserve, showing that AMH and FSH levels did not vary between women with vitamin D deficiency and those with normal levels (0.8 ± 3.0 vs. 0.5 ± 1.6 ng/mL (*p* = 0.18) and 9.4 ± 7.2 vs. 9.2 ± 9.5 mIU/mL (*p* = 0.54), respectively) [57]. Multivariate linear regression analysis of log-transformed AMH and FSH with 25OH-D levels adjusted for age, BMI, and seasonal variation confirmed a lack of association [57]. In a small case-control study by Lata et al. [56], women with unexplained infertility were compared to fertile controls in terms of AMH and vitamin D levels. Vitamin D deficiency was present in 64.28% of infertile females. In vitamin D-deficient cases, the mean for vitamin D was 6.18 ± 2.09, and AMH was 1.94 ± 1.30. In vitamin D-deficient controls, the mean for vitamin D was 4.85 ± 3.02, and AMH was 3.47 ± 2.59. On comparison, the vitamin D levels were lower in fertile than infertile females, which was significant (*p* = 0.04), and AMH levels were lower in cases than the control group. No correlation between AMH and vitamin D was found in either group. However, no actual values were reported, and it should be noted that all women were vitamin D-deficient, and therefore, the relationship between vitamin D and AMH could not be evaluated across the full vitamin D level spectrum. Moreover, this study did not control for potential confounders such as age, BMI, and smoking. Similarly, Neville et al. [54] found no significant correlation between 25-OH-D and AMH in their study that examined the relationship between vitamin D and various fertility factors among female and male partners undergoing IVF/ICSI (intracytoplasmic sperm injection). Women (*n* = 64) undergoing a fresh IVF cycle provided blood samples on the day of oocyte retrieval. Vitamin D levels were not significantly correlated with AMH (*p* = 0.629) [54]. Another study conducted by Bednarska-Czerwińska et al. [58] comprised 53 women diagnosed with tubal factor infertility. Using Pearson’s linear correlations, they found overall a nonsignificant negative correlation between serum AMH and total vitamin D (r = −0.19, CI 95%: (−0.46, 0.12), *p*= 0.22). Interestingly, a change-point in the relationship was noted: a negative linear correlation between levels of serum AMH and total vitamin D concentrations up to approximately 31 ng/mL (*p* = 0.06); beyond that threshold, a nonsignificant positive correlation was observed (*p* = 0.50). The authors also found a significant, negative correlation between follicular fluid AMH and total vitamin D (r = −0.28, CI 95%: (−0.51, 0.02), *p* = 0.0391). In addition, they investigated seasonal differences in various biomarkers and found fluctuations between winter/spring and summer/autumn in serum vitamin D and serum AMH levels (*p* = 0.0363 and *p* = 0.0165, respectively) [58]. Another study conducted by Liu et al. [59] involved a large cohort of women (*n* = 848) that had indications for IVF. Like Bednarska-Czerwińska et al., Liu et al. found seasonal fluctuations in serum vitamin D levels, with levels being the highest in autumn. Serum vitamin D levels were inversely related to AMH and BMI, although these findings were statistically insignificant (*p* = 0.134 and 0.099, respectively) [59].

#### 3.3.2. Interventional Studies

There have been four prospective clinical studies and two randomized placebo-controlled trials that evaluated the effects of vitamin D supplementation on serum AMH levels in women, the data from which is summarized in Table 2. In a prospective study of 33 women in New Zealand between the ages 19–39 in 2012, Dennis et al. investigated the effects of vitamin D supplementation on serum AMH levels over a six-month period [61]. Participants had baseline blood measurements of AMH and vitamin D levels in the summer and were then given daily doses of 1000 IU vitamin D3 (*n* = 16), 1000IU vitamin D2 (*n* = 7), or a placebo (*n* = 10) over the autumn and winter months. Both serum 25OH-D and AMH levels showed seasonal variations, with an 18% decrease in AMH levels in winter compared to the summer baseline levels. Interestingly, this study demonstrated that 1,25-dihydroxyvitamin D3 supplementation, but not a placebo or vitamin D2 treatment, was able to block the seasonal decline in both 25OH-D and AMH levels (61). There was a strong correlation between the change in vitamin D levels (Δ25OH-D) and the change in AMH levels (ΔAMH) (r = 0.36, *p* = 0.004). Limitations of this study include its relatively small female population size, as well as the possibility that the observed decline in AMH was related to natural aging (over six months) rather than a seasonal variability in vitamin D levels between summer to winter. The same investigators subsequently conducted a randomized double-blind clinical trial in young women (18–25 years old) with regular menstrual cycles recruited from the community to evaluate the effects of an acute rise of vitamin D on systemic AMH levels over a one-week period [62]. The study was conducted in the early spring when vitamin D levels tend to be at their nadir. The women received either an oral dose of 50,000 IU of vitamin D3 (*n* = 27) or a placebo (*n* = 22) once weekly. The authors reported that women receiving vitamin D3 treatments showed robust increases in serum 25OH-D within one day of treatment (15.8 ± 1.1 nmol/L, *p* < 0.0001), followed by a progressive rise in AMH levels during the following week, with a mean increase of 12.9% ± 3.7% (*n* = 24, *p* = 0.001). This was in contrast to the placebo group, in which no significant changes in AMH levels were observed [62]. In a study of 30 infertile Iranian women >35 yo with regular cycles but diminished ovarian reserves (as defined by AMH < 0.7 ng/mL) and vitamin D deficiencies or insufficiencies (levels <30 ng/mL), Naderi et al. similarly reported a significant increase in serum AMH levels (from 0.39 ± 0.26 to 0.92 ± 0.62 ng/mL, *p* < 0.000) following three months of 50,000 IU oral vitamin D3 supplementations. In addition, there was a significant positive correlation between the serum levels of 25OH-D posttreatment and AMH levels, with mean AMH levels after treatment being higher in women with sufficient 25OH-D vs. women with insufficient 25OH-D (1.048 ± 0.644 vs. 0.513 ± 0.284 ng/mL (*p* = 0.043) [63].

Three interventional studies specifically evaluated women with PCOS. Irani et al. investigated the effects of vitamin D supplementations on AMH levels in a prospective study of 67 US women with (*n* = 2 2) or without (*n* = 4 5) PCOS who were diagnosed with vitamin D deficiency (<2 0 ng/mL) [64). Fifty-one women were replaced with 50,000 IU oral vitamin D3 for eight weeks (16 with PCOS and 35 controls), and 16 women were not treated (six with PCOS and 10 controls). Vitamin D supplementations were effective in both PCOS and non-PCOS treated groups, resulting in the normalization of vitamin D serum levels following eight weeks of treatment. The study showed that the 25-dihydroxyvitamin D3 supplementations resulted in a decrease in serum AMH levels only in the PCOS population, while no change in AMH level was observed in non-PCOS women following vitamin D supplementations [64]. In a randomized, double-blind trial consisting of only PCOS women, Dastorani et al. [66] also showed that vitamin D supplementations reduced serum AMH levels. Women received either 50,000 IU vitamin D or a placebo (*n* = 17/group) every other week for eight weeks. Compared with baseline levels, end-of-trial serum AMH levels were significantly reduced in the vitamin D group compared with the placebo group (−0.7 ± 1.2 vs. −0.1 ± 0.5, *p* = 0.02). Not only did vitamin D supplementations affect AMH, but it also led to decreased insulin levels (−1.4 ± 1.6 vs. −0.3 ± 0.9, *p* = 0.007) and decreased total cholesterol levels compared with the placebo (−5.1 ± 1 2.6 vs. 2.9 ± 1 0.9, *p* = 0.03), respectively [66]. Limitations of this study included the smaller sample size, as well as the shorter duration of intervention. It is also important to note that all participants were vitamin D-deficient from the beginning, perhaps limiting the scope of the results. In contrast to the above studies, Cappy et al. reported no changes in serum AMH levels following vitamin D supplementations [65]. Their study included 50 vitamin D-deficient women with either PCOS (*n* = 23) or with normal ovarian reserves (*n* = 27) from France. Vitamin D supplementations were provided differentially according to the severity of deficiency; the duration of the treatment was two weeks for the light deficiency (25OH-D between 29 to 20 ng/mL), four weeks for the moderate deficiency (25OH-D between 19 to 10 ng/mL), and six weeks for the deep deficiency (25OH-D < 10 ng/mL). Both PCOS and normal ovarian reserve groups had significant increases in vitamin D levels following the supplementations. However, in contrast to the studies by Irani et al. and Dastorani et al., no differences in serum AMH levels before and after treatments were observed in either patient group. In both groups, 25OH-D serum levels were not related to serum AMH levels [65].

### 3.4. Meta-Analysis of Interventional Studies

#### 3.4.1. Effects of Vitamin D Supplementation on Serum 25OH-D Levels

**Figure 2:**
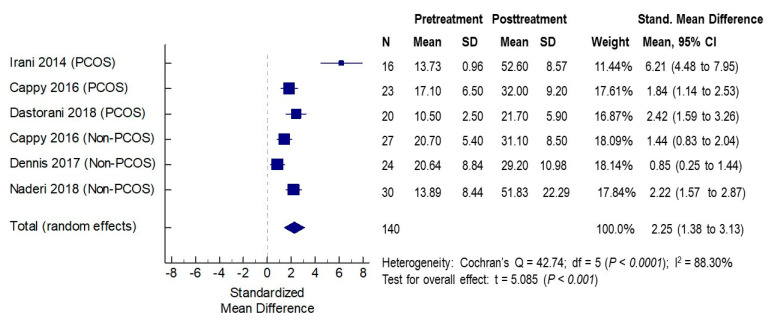
Effects of vitamin D supplementation on serum vitamin D levels. PCOS: polycystic ovarian syndrome. CI: confidence interval; df: degrees of freedom.

In the five studies included in the meta-analysis, the effects of vitamin D supplementation on serum 25OH-D concentrations were assessed in six cohorts of patients (three PCOS and three non-PCOS), a total of 140 women. The baseline mean serum vitamin D concentrations across studies ranged from 10.5 to 20.7 ng/mL pretreatment, indicating that the majority of women had vitamin D deficiency. The mean serum concentrations post-vitamin D treatments ranged from 21.7 to 52.6 ng/mL. In all studies, significant increases in serum vitamin D levels were noted following vitamin D supplementations, with SMD ranging from 0.84 to 6.21, indicating the efficacy of vitamin D supplementations in all studies. The overall standardized mean difference (SMD) was 2.25 (95% CI 1.38 to 3.13, *p* < 0.001) (Figure 2).

#### 3.4.2. Effects of Vitamin D Supplementation on Serum AMH Levels

**Figure 3:**
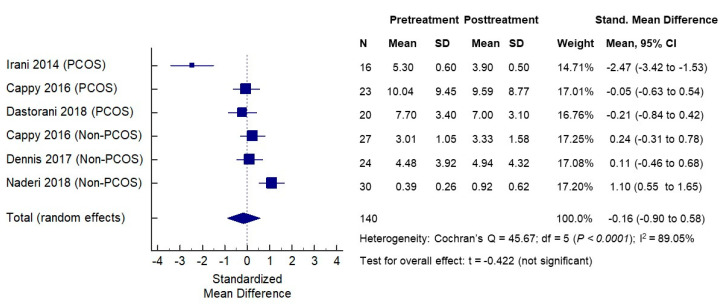
Effects of vitamin D supplementation on serum AMH levels—all women.

**Figure 4:**
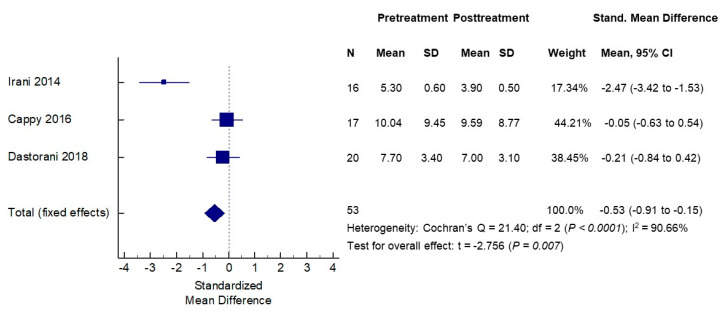
Effects of vitamin D supplementation on serum AMH levels—PCOS women.

**Figure 5:**
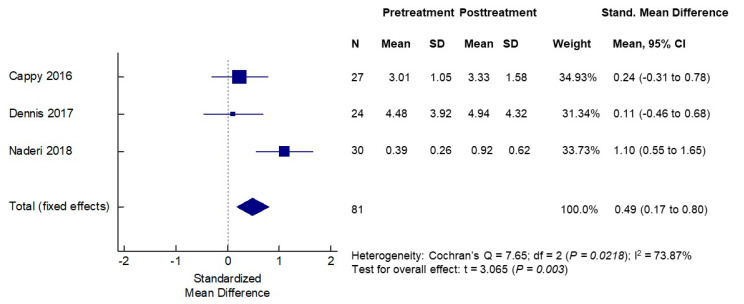
Effects of vitamin D supplementation on serum AMH levels—non-PCOS women.

The effects of vitamin D supplementation on serum AMH concentrations were assessed in the same six cohorts of patients (three PCOS and three non-PCOS), a total of 140 women. The baseline mean serum AMH levels in the PCOS groups ranged from 5.30 to 10.04 ng/mL and, as expected, were higher than the mean serum AMH levels in non-PCOS groups that ranged from 0.39 to 4.48 ng/mL. The mean serum AMH concentrations post-vitamin D treatments ranged from 3.90 to 9.59 ng/mL in the PCOS cohorts, while they ranged from 0.92 to 4.94 ng/mL in the non-PCOS groups. When analyzing PCOS and non-PCOS cohorts together, no significant changes in serum AMH levels were observed following vitamin D supplementations (SMD −0.16, 95% CI −0.90 to 0.58) (Figure 3). However, significant opposite changes in serum AMH levels posttreatment were noted when the PCOS and non-PCOS cohorts were analyzed separately. In the meta-analysis of three PCOS cohorts (*n* = 53 women), the serum AMH was significantly decreased following vitamin D supplementation (SMD −0.53, 95% CI −0.91 to −0.15, *p* < 0.007) (Figure 4). In contrast, in the meta-analysis of three non-PCOS cohorts (*n* = 81), serum AMH was significantly increased following vitamin D supplementation (SMD 0.49, 95% CI 0.17 to 0.80, *p* = 0.003) (Figure 5).

## 4. Discussion

The current systematic review included eighteen cross-sectional studies and six interventional studies assessing the relationship between serum vitamin D levels and serum AMH levels. The cross-sectional studies demonstrated conflicting results, with several studies finding a positive correlation between serum AMH and vitamin D levels [42,46,50,61], while most studies reported largely negative findings [43,44,45,47,49,51,52,53,54,55,56,57,58,59]. The systematic review and meta-analysis of the interventional studies revealed that vitamin D supplementations affect serum AMH levels but have opposite effects depending on the ovulatory statuses of the women. It increased serum AMH levels in ovulatory non-PCOS women, while it decreased AMH levels in PCOS women.

There can be several reasons underlying the discrepant findings in the cross-sectional studies. Heterogeneity in the populations studied may account for some of the conflicting data reported, as some studies looked at normal noninfertile ovulatory women, while others looked at women with PCOS or with diminished ovarian reserve (DOR) and others specifically at infertile women (Table 1). Moreover, individual vitamin D levels are influenced by race/ethnicity, geographic area, and seasonality (sun exposure) and have been suggested to play a role in ovarian reserve differences [67]. In particular, the range of serum vitamin D levels in a given population varies widely between studies such that the proportions of individuals with vitamin D deficiencies, insufficiencies, or replete statuses vary widely between studies. This may be a particularly important source of variation in reported findings between studies, since the relationship between vitamin D and AMH has been suggested to be nonlinear. In fact, Bednarska-Czerwińska et al. [58] reported a negative correlation between AMH and vitamin D concentrations of up to approximately 30 ng/mL, while beyond that concentration, the trend became positive but statistically insignificant. In addition, differences in the population size may also account for negative findings, as some of the studies had small numbers of patients [43,49,51,52,53,54,56,58] and were not adequately powered to detect small but significant associations between vitamin D and AMH.

Cross-sectional studies are inherently limited by their nature of evaluating a single time point, which does not take into account individual fluctuations in AMH and vitamin D, as well as potential seasonal variations. Moreover, these studies do not allow to determine the nature of the association (i.e., causality). Therefore, more insight regarding the relationship between serum vitamin D and AMH, as well as the potential causality, can be gained from prospective interventional studies.

Among the six interventional studies included in this systematic review, three out of six studies reported an increase in serum AMH levels in non-PCOS vitamin D-deficient women following both acute [62] and long-term [61,63] vitamin D supplementations (Table 2). In contrast to these three studies, Cappy et al. found no changes in serum AMH following vitamin D supplementations in either PCOS or non-PCOS women. A possible reason for this discrepancy may be related to the efficacy of vitamin D supplementations, since the mean posttreatment vitamin D levels in the study by Cappy et al. were just above the insufficiency level (31.1 ± 8.5 and 32.0 ± 9.2 ng/mL in the control and PCOS women, respectively), much lower than the other studies.

Two other interventional studies that included PCOS women by Irani et al. and Dastorani et al. showed that vitamin D supplementations led to decreases in AMH levels only in women with PCOS [64,66]. Importantly, our meta-analysis revealed that vitamin D supplementations led to significant increases in serum AMH in non-PCOS women, whereas it led to significant decreases in serum AMH in PCOS women. While these results may appear conflicting at first, one can reconcile them by considering the context of the different follicular environment and AMH statuses of women with PCOS. Women with PCOS characteristically have abnormally high serum AMH levels, which are reflective of the quantity of their numerous arrested small ovarian antral follicles [68]. The increased AMH levels in their cases correlate with the severity of PCOS manifestations (amenorrhea and hyperandrogenism) [69]. Various treatments for PCOS, including metformin [70,71,72], clomiphene citrate [73], and laparoscopic ovarian drilling [74,75], have been shown to result in decreased serum AMH levels associated with improvements in clinical symptoms. Vitamin D supplementations have also been shown in various studies to lead to improvements in clinical manifestations of PCOS [76,77], and thus, an associated decrease in AMH is not surprising but, rather, likely reflects the improvement in folliculogenesis and ovulatory statuses in these women. Interestingly, Szafarowska et al. recently reported that polymorphisms Fok1 (rs2228570) and Apa1 (rs7975232) in the vitamin D receptor (VDR) gene are associated with elevated AMH levels in PCOS [52], supporting the notion that vitamin D may influence AMH levels in PCOS. It is possible that such genetic variations contribute to differences in the findings on the association between vitamin D and AMH levels among different studies. It would be interesting to further explore whether the effects of vitamin D supplementations on serum AMH levels may be different depending on such genetic variations.

## 5. Conclusions

There is substantial evidence from both animal and human studies suggesting a role for vitamin D in female reproductive physiology [14]. The existence of vitamin D-responsive elements on the AMH gene promoter provides a clear scientific basis for vitamin D to exert its effects on AMH gene expression levels, which is supported by in vitro animal and human studies. However, cross-sectional studies have reported largely conflicting findings regarding an association between serum vitamin D and AMH levels. These discrepant findings are likely due to the heterogeneity in study populations, as well as the apparently complex nonlinear relationship that exists between vitamin D and AMH, as was recently suggested [58]. In contrast, results from most interventional studies that examined the effects of vitamin D supplementations on serum AMH levels indicate a cause-effect relationship between vitamin D and AMH. Our meta-analysis provides evidence that the direction of vitamin D effects on AMH appears to be dependent on a woman’s ovulatory status. Interestingly, vitamin D supplementations appear to decrease AMH levels in anovulatory women with PCOS, while vitamin D supplementations appear to increase AMH levels in ovulatory non-PCOS women. In PCOS, in which AMH is abnormally elevated, this likely reflects the ability of vitamin D to improve folliculogenesis and is consistent with evidence from numerous clinical trials showing improved clinical manifestations following vitamin D supplementations. Importantly, since AMH is produced by granulosa cells of preantral and small antral follicles, but not primordial follicles, it is merely a proxy of the “ovarian reserve”. Therefore, it remains to be determined whether any changes in AMH levels induced by vitamin D supplementations may be reflective of local folliculogenesis changes in the ovarian microenvironment or, rather, may be indicative of true alterations in a woman’s ovarian reserve. Nonetheless, vitamin D is an oral, relatively safe and inexpensive supplement, and accumulating evidence suggests its potential benefit for various aspects of human reproduction, including increased rates of pregnancy and live births in assisted reproductive technology [78], decreasing the risk of pregnancy loss [77], as well as various pregnancy complications [79]. Large, randomized trials of vitamin D supplementations focusing on different vitamin D status ranges are necessary to gain more insight into the complex nature of the relationship between vitamin D and ovarian reserve markers and its potential benefit to human reproduction in general.

## Figures and Tables

**Table 1:** Observational studies.

Study	Country	Population	Exclusion Criteria	Age, Years (Range, Mean or Median)	Study Design	AMH Assay	Relationship between Vitamin D and AMH	Covariates Adjusted	Vitamin D Status
**Healthy/Normal Menstrual Cycle**									
Merhi 2012 [42]	USA	Premenopausal women (*n* = 388) with regular menstrual cycles who were either HIV positive or high-risk HIV negative	Cancer; hepatic disease; prior hysterectomy/oophorectomy; pregnant	25–45	Cross-sectional	DSL ELISA	Serum 25OH-D was positively correlated with serum AMH levels in late-reproductive-age women (≥40 years old); a weak negative correlation between serum vitamin D and AMH was noted in young individuals (<35 years of age)	HIV status BMI Race Smoking Current drug use Fasting Glucose Insulin level EGFR Geographic site	14 (9–21) ng/mL
Chang 2014 [43]	South Korea	73 healthy women	Obesity; history of infertility; systemic disease; smoking; medication or nutritional supplements in last 3 months; irregular menstrual cycles	27–38	Cross-sectional	Gen II ELISA	25(OH)D did not correlate with AMH or other ovarian reserve markers	None	Baseline 25(OH)D concentration of the study population (in winter): 10.3 +/− 4.6 ng/mL
Fabris 2017 [44]	Spain	Healthy oocyte donors (*n* = 851) with regular menses and at least six antral follicles per ovary divided into 3 groups according to vitamin D level (<20, 20–30, >30 ng/mL)	PCOS women	18–35	Cross-sectional	Elecsys automated assay (Roche)	No correlation between serum AMH and bioavailable vitamin D; no correlation between AFC and bioavailable vitamin D	Age BMI	29.5% were vitamin D replete (>30 ng/mL); 52% had vitamin D deficiency (20–30 ng/mL), and 18.5% had insufficient vitamin D (<20 ng/mL)
Kim 2018 [45]	South Korea	291 premenopausal women with regular period	Hysterectomy and ovarian surgery; chemotherapy or radiation; estrogen suppressive therapy; OCPs; medications; calcium or vitamin D supplements; abnormal thyroid function test; undetectable AMH	35–49	Cross-sectional	AMH Gen II	There was no correlation between AMH and 25OH-D after adjustment for age.	Age	76.6%, of subjects were serum vitamin D-insufficient (<20 ng/mL); mean vitamin D level 15.9 ng/mL
Jukic 2018 [46]	USA	825 women aged 30 to 44 years without any known fertility problems	Hx of infertility PCOS Endometriosis Partner with infertility Recurrent breastfeeding	30–44	Cross-sectional	ELISA (ANSH Labs)	25(OH)D was not correlated with AMH, FSH, or inhibin-B. Multivariable results with continuous hormonal outcomes were also null. For dichotomous outcomes, there was a tendency for insufficient 25(OH)D (<30 ng/mL) to be associated with low AMH (<0.7 ng/mL)	Age, Race, BMI, Smoking Hx, Recent use of birth control	36 ± 11 ng/mL
Purdue-Smith 2018 [47]	USA	US registered nurses who participated in the NHS2 prospective study (1989): women who experienced menopause between time of blood collection and age 45 (cases, *n* = 328), women who experienced menopause after age 48 (controls, *n* = 328)	Cancer; Cardiovascular disease; hysterectomy or oophorectomy; radiation or chemotherapy-induced menopause; menopause prior to blood draw	32–54	Cross-sectional	Pico AMH assay (ANSH Labs)	Adjusted geometric means of AMH concentrations did not vary according to free 25(OH)D concentration quartiles or total 25(OH)D concentration quartiles	Age; Smoking; BMI; Parity; Physical activity; Breastfeeding; Timing of blood collection; Alcohol intake; Dietary intake;	Quartiles for total 25(OH)D concentrations (nmol/L) and number of cases:controls Q1, median 44.2, 86:83 Q2, median 59.8, 92:83 Q3, median 71.1, 71:82 Q4, median 90.4, 79:80
Xu 2019 [48]	China	33 women with POI, no iatrogenic cause or chromosomal abnormality, and no hormone therapy for at least 6 months; 72 healthy women with regular menstrual cycles and no history of infertility	Taking vitamin D supplements or other medications that affect vitamin D and ovarian reserve determinants; hysterectomy; oophorectomy; ovarian surgery; chemotherapy or radiation; cigarette smoking; autoimmune disease	18–40	Cross-sectional	Electrochem-iluminescence immunoassay (Cobas e602)	25(OH)D levels were positively but insignificantly correlated with log-transformed AMH, even after adjusting for confounders	Age BMI Education Annual household income	POI women: 25OH-D: 92.38 +/− 31.07 nmol/L Control women 25OH-D: 96.76 +/− 33.12 nmol/L
**Women with polycystic ovary syndrome**									
Pearce 2015 [49]	Australia	PCOS (*n* = 58) and non-PCOS (*n* = 282) women presenting to fertility clinic	Undetectable serum AMH levels (<3 pmol/L); Consumption of supplements containing more than 500 IU of vitamin D	<40	Cross-sectional	ELISA (Immunotech)	Seasonal variations in serum vitamin D were observed between summer and winter, but no seasonal variation in serum AMH levels; no correlation between serum AMH or AFC and vitamin D levels over the year even after adjustment for known confounders. When the cohort was divided into PCOS and ovulatory groups, still no significant relationship was observed.	Age, BMI, Skin color, Menstrual cycle length, Occupation	Summer month 83.4 +/− 5.9 nmol/L Winter month 49.3 +/− 3.6 nmol/L
Wong 2018 [50]	Hong Kong	451 PCOS women and 244 healthy ovulatory women	Active endocrine or metabolic disease; using any prescription drug; pregnant in the past 3 months	18–40	Cross-sectional	Chem-iluminescent immunoassays	Both serum 25(OH)D and AMH levels peaked during summer in PCOS women. In ovulatory women, only serum 25(OH)D but not AMH level showed such seasonal variation. Serum 25(OH)D level in PCOS women significantly correlated positively with AMH, AMH/AFC ratio, and other metabolic parameters; 25(OH)D level was an independent predictor of serum AMH level in women with PCOS but not in ovulatory women.	Age BMI Free androgen index	74.9% Vitamin D deficient (<20 ng/mL); 21.7% vitamin D insufficient (between 20–29 ng/mL) 3.3% vitamin D sufficient (>30 ng/mL)
Bakeer 2018 [51]	Egypt	53 PCOS females with infertility and 17 healthy controls	Cushing syndrome, androgen-secreting tumors, congenital adrenal hyperplasia and hyperprolactinemia	17–39	Cross-sectional	ELISA or colorimetric	No significant correlation existed between AMH and 25(OH)D, BMI, and dyslipidemia markers.	Age	PCOS 31.32 ± 14.85 (nmol/L) Control 48.65 ± 27.30 (nmol/L)
Szafarowska 2019 [52]	Poland	25 patients with PCOS and 23 control women	Women on oral hormonal contraception; hormonal intrauterine device	25–43	Cross-sectional	DRG ELISA EIA-5738	There was no correlation between AMH and 25(OH)D levels in the PCOS or in the control group. Genetic analysis revealed associations between VDR polymorphisms and AMH levels in PCOS women.	Age	Vitamin D levels in PCOS group (14.2 ng/mL) were lower than control group (19.6 ng/mL)
Arslan 2019 [53]	Turkey	146 infertile women divided into normal ovarian reserve (*n* = 86) vs. high ovarian reserve (PCOS, *n* = 60). Women were further divided based on VDD: (Group A) severe VDD (<10 ng/mL, *n* = 101) and (Group B) mild VDD (10–20 ng/mL, *n* = 45)	Smoking; prior hysterectomy and/or oophorectomy; endometriosis; ovarian masses; menopause; pregnancy; endocrine disorders; renal dysfunction; hypertension	18–35	Cross-sectional	ECLIA method using an automated analyzer (Cobas 6000)	Serum AMH levels were not correlated with 25(OH)D levels in the normal ovarian reserve or PCOS group	Age BMI FSH LH Steroid hormones	Normal ovarian reserve group 25(OH)D 9.0 ± 6.0 (ng/mL) PCOS group 25(OH)D) 8.5 ± 6.7 (ng/mL
**Infertile/IVF women**									
Neville 2016 [54]	Ireland	Couples using their own gametes for a fresh IVF/ICSI cycle (*n* = 64 women)	None	36.5 ± 3.3	Cross-sectional	Not reported	No significant correlation between 25(OH)D and AMH	None	Mean serum 25(OH)D concentration 47.4 ± 2.8 nmol/L; 12 deficient (<30 nmol/L), 28 suboptimal (30–50), 24 sufficient (>50)
Drakopoulos 2017 [55]	Belgium	Healthy infertile women (*n* = 283) undergoing their first infertility treatment divided into vitamin D deficient (<20 ng/mL or normal vitamin D levels (≥20 ng/mL)	Vitamin D supplementation; medication for systemic disease; iatrogenic (ovarian sx., gonadotoxic therapy) or genetic cause of ovarian reserve loss	18–42	Cross-sectional	Gen II ELISA	The mean AMH and AFC levels did not differ significantly between the vitamin D-deficient and the vitamin D-normal groups; No correlation was observed between 25-OH Vitamin D and AMH or AFC	Age BMI Infertility cause Smoking Season	30.7% (*n* = 87) had vitamin D < 20 ng/mL; 69.3% (*n* = 196) had vitamin D >20 ng/mL
Lata 2017 [56]	India	Infertile women with unexplained infertility (*n* = 35) and fertile controls (*n* = 35). Both groups were vitamin D-deficient	History of smoking; OCPs; any hormonal or steroid drug use; known VDD, obesity (BMI > 35); endometriosis; thyroid disorders; autoimmune disease; tubal factor, male factor, or PCOS	18–40	Cross-sectional	ELISA (ANSH labs)	No correlation between AMH and Vitamin D was found in either group (no values reported).	Age, duration of married life, duration and type of infertility, obstetrical history, education level	Case 6.18 ± 2.09 ng/mL Control 4.85 ± 3.02 ng/mL
Shapiro 2018 [57]	USA	457 infertile women with high prevalence of diminished ovarian reserve	All women who had baseline measurements of 25OH-D, AMH, and FSH within 90 days of each other were included	21–50	Cross-sectional	Not reported	AMH and FSH levels did not vary between women with VDD and those with normal levels; Multivariate linear regression analysis of log-transformed AMH and FSH with 25OH-D levels adjusted for confounders confirmed lack of association.	Age BMI Seasonal variations	16.2% (*n* = 74) had 25OH-D <20.0 ng/mL; 83.8% (*n* = 383) had 25OH-D ≥20 ng/mL
Bednarska-Czerwińska 2019 [58]	Poland	53 infertile women (diagnosed with tubal factor infertility and qualified for IVF) with AMH >0.7 ng/mL	Hypertension; diabetes; renal dysfunction; hyperinsulinism; PCOS; endometriosis	34.7 ± 4.1	Cross-sectional	ECLIA immunoanalyzer (Cobas e411)	Overall, a nonsignificant negative linear correlation between serum AMH and total vitamin D; However, a change-point was noted; Negative linear correlation between levels of serum AMH and total vitamin D concentrations up to approximately 31 ng/mL; Beyond that threshold, a nonsignificant positive correlation was observed.	Age BMI	Total vitamin D (ng/mL) in serum overall: 29.7 ± 13.3; During winter/spring: 26.3 ± 13.2; During summer/autumn: 34.2 ± 12.6
Liu 2019 [59]	China	848 infertility patients undergoing IVF	Patients with premature ovarian insufficiency; patients treated with ICSI; women whose 25(OH)D levels were taken 4 weeks prior to IVF cycle	31.67	Cross-sectional	Not reported	Serum Vitamin D levels were inversely related to AMH, although this was not statistically significant.	None	Patients divided into 4 groups based on serum 25(OH)D quartiles (ng/mL); Group 1: 9.04; Group 2: 13.67; Group 3: 16.20; Group 4: 23.22

AFC, antral follicle count; AMH, anti-Müllerian hormone; AUC, area under the curve; BMI, body mass index; ELISA, enzyme-linked immunosorbent assay; EGFR, estimated glomerular filtration rate; FSH, follicle-stimulating hormone; Hx, history; LH, Luteinizing hormone; ICSI, intracytoplasmic sperm injection; 25OH-D, 25-hydroxy vitamin D; OCP, oral contraceptive pills; PCOS, polycystic ovary syndrome; POI, premature ovarian insufficiency; ROC, receiver-operating characteristic curve; VDD, vitamin D deficiency; NHS2, Nurses’ Health Study II; and IVF, in vitro fertilization.

**Table 2:** Interventional studies.

Study	Country	Population	Exclusion Criteria	Age (Range, Mean or Median)	Study Design	Intervention	AMH Assay	Effects of Intervention on Serum AMH	Factors Adjusted	Vitamin D Level
Dennis 2012 [61]	New Zealand	Women (*n* = 33) were given a daily vitamin D or placebo for 6 months	Women near menopause (>40) or an AMH <0.5 ng/mL; a seasonal change in weight > 4 kg, or a BMI > 25 kg/m^2^	19–39	Prospective	Daily supplements of either 1000 IU ergocalciferol (D2) (*n* = 7) or 1000 IU of cholecalciferol (D3) (*n* = 16) or placebo (*n* = 10) for 6 months (summer to winter)	DSL ELISA or Gen II	Change in AMH level correlated with the initial AMH level and the magnitude of change in vitamin D levels (r = 0.36, *p* = 0.004). Vitamin D supplementation prevented seasonal AMH change.	Age BMI	Not listed
Irani 2014 [64]	USA	PCOS (*n* = 22) and non-PCOS (*n* = 45) women with vitamin D deficiencies	Pregnant, postpartum, or breastfeeding; exogenous hormones; any form of oral vitamin D3 replacement; poor ovarian reserve	PCOS: Nontreated 31.3 +/− 3.1 vs. treated 27.0 +/− 0.9 Non-PCOS: Nontreated 28.5 +/−1.5 vs. treated 28.7 +/− 1.3	Prospective	Sixteen of the 22 women with PCOS and 35 of the 45 controls were treated with 50,000 IU of vitamin D3 orally once weekly for 8 weeks	Gen II ELISA	In women with PCOS, vitamin D3 supplementation was associated with a decrease in serum AMH levels. There was no significant change in AMH levels after vitamin D3 replacement among women without PCOS	Age, BMI, Race, Skin color, h/o DM, h/o infertility, smoking, daily milk consumption	Non-PCOS women: 25OH-D increased from 13.31 ± 0.37 to 42.32 ± 3.67 (ng/mL) after treatment; PCOS: 25OH-D changed from 13.73 ± 0.96 to 52.60 ± 8.57 after treatment
Cappy 2016 [65]	France	PCOS (*n* = 23), and normal ovarian reserve (NOR) women (*n* = 27) with vitamin D deficiency	Contraindication to a standard vitamin D supplementation (sarcoidosis, renal insufficiency, allergy); Having received vitamin D supplementation in prior 3 months	NOR 30.8 ± 5.4 PCOS 27.1 ± 4.4	Prospective	Vitamin D supplementation was given according to severity of deficiency: 2 vials of 100,000 IU over 2 weeks if 25(OH)D between 20–29 ng/mL; 3 vials over 4 weeks if 25(OH)D between 10–19 ng/mL, and 4 vials over 6 weeks if 25(OH)D < 10 ng/mL	Immunotech ELISA	No difference in serum AMH levels before and after treatment was observed either in PCOS patients or in NOR patients. In both groups, 25(OH)D serum levels were not related to serum AMH levels, serum 1,25(OH)2D, and serum PTH levels, before and after treatment.	Age BMI	Controls: Vitamin D level before treatment 20.7 ± 5.4 vs. after treatment 31.1 ± 8.5 ng/mL PCOS: Vitamin D level before treatment 17.1 ± 6.5 vs. after treatment 32.0 ± 9.2 ng/mL
Dennis 2017 [62]	New Zealand	Women (*n* = 49) with regular menstrual cycle recruited from the community	Pregnant; recent breastfeeding; Vitamin D supplementation; traveled to the Northern hemisphere, sunbeds use; any history of endocrine or reproductive diseases, including PCOS	Control 21.7 ± 1.4 (19.4–25.2) Vitamin D3 21.7 ± 1.1 (19.6–24.5)	Randomized, double-blinded, placebo-controlled trial	50,000 IU Vit D orally once weekly (*n* = 27) or placebo (*n* = 22)	Gen II ELISA	All women receiving vitamin D3 treatment exhibited a robust increase in serum 25(OH)D within 1 day; Circulating levels of AMH in the Vitamin D3 group progressively rose with a mean increase of 12.9 ± 3.7% but not in the control group.	Age BMI Asthma medication Oral contraceptive	Control: Baseline vitamin D level 54.1± 25.9 nmol/L (16.1–115) Vitamin D3 group: Baseline vitamin D level 51.6 ± 22.1 (9.7–109)
Naderi 2018 [63]	Iran	Infertile women (*n* = 30) with diminished ovarian reserve (AMH <0.7 ng/mL) but regular menstrual cycle with vitamin D insufficiency or deficiency	Any systemic disorder; PCOS; vitamin D supplementation; using any hormonal therapies; smoking; any malignancies; hysterectomy and oophorectomy; pregnancy.	>35	Prospective	50,000 IU Vitamin D orally once weekly for 3 months	Elecsys automated assay (Roche)	Vitamin D supplementation resulted in a significant increase in serum AMH levels (from 0.39 ± 0.26 to 0.92 ± 0.62 ng/mL. There was a significant positive correlation between serum levels of 25(OH) D posttreatment with AMH level	Age BMI fertility	Mean posttreatment level of 25OH-D in women with AMH >0.7 ng/mL (*n* = 19 women) was higher than that in women with AMH <0.7 ng/mL (*n* = 11 women) (59.332 ± 21.751 vs. 38.881 ± 17.281 ng/mL)
Dastorani 2018 [66]	Iran	40 infertile women with PCOS	Metabolic disorders; thyroid disorders; diabetes or impaired glucose tolerance	18–40 Placebo group: 30.1 ± 3.4 Vitamin D group: 29.9 ± 4.4	Randomized, double-blinded, placebo-controlled trial	Women received 50,000 IU vitamin D (*n* = 17) or placebo (*n* = 17) every other week for 8 weeks (6 lost to follow-up)	ELISA kit	Vitamin D supplementation led to a significant decrease in serum AMH (−0.7 ± 1.2 in vitamin D treatment group vs. −0.1 ± 0.5 ng/mL in placebo)	Age Height Weight BMI	Placebo group: Baseline vitamin D 11.0 ± 2.4. vs. posttreatment 10.9 ± 2.1 ng/mL Supplementation group: Baseline vitamin D 10.5 ±2.5 vs. posttreatment 21.7 ± 5.9 ng/mL

AMH, anti-Müllerian hormone; BMI, body mass index; DM. diabetes mellitus; ELISA, enzyme-linked immunosorbent assay; 25OH-D, 25-hydroxy vitamin D; h/o, history of; NOR, normal ovarian reserve; OCP, oral contraceptive pills; PCOS, polycystic ovary syndrome; PTH, parathyroid hormone.

## Supplementary Materials

The following are available online at https://www.mdpi.com/2072-6643/12/6/1567/s1, Table S1: Newcastle-Ottawa Scale (NOS) for assessing quality of interventional cohort studies.
