# Supplementary material for: The Association between Vitamin D and Anti-Müllerian Hormone: A Systematic Review and Meta-Analysis
Source: Nutrients. 2020 May 28;12(6):1567. doi: 10.3390/nu12061567 (PMC7352921; doi:10.3390/nu12061567)
Supplement: Supplementary file 1 [file nutrients-12-01567-s001.pdf]

nutrients

MDPI

Supplementary Table S1. Newcastle-Ottawa Scale for assessing quality of interventional cohort studies.

		Selection of study groups				Compara-bility	Outcome			
Author	Year	Represent-ativeness of exposed cohort	Selection of non-exposed cohort	Exposure assessment	Outcome of interest not present at start of study	Compara-bility of cohorts	Outcome assessment	Adequacy of length of time before follow-up	Adequacy of follow-up of cohorts	Total quality scores
Irani, M	2014	★	★	★	★	★★	★	★	★	9
Cappy, H	2016	★	★		★	★★	★	★	★	8
Dennis, NA	2017	★	★	★	★	★★	★		★	8
Naderi, Z	2018	★	★	★	★	★★	★	★	★	9
Dastorani, M	2018	★	★	★	★	★★	★	★	★	9

[a] A study can be awarded a maximum of one star for each numbered item except for the item, “Comparability of cohorts”. [b] A maximum of two stars can be awarded for “Comparability of cohorts”.
